# T192. THE GUT MICROBIOME IN SCHIZOPHRENIA AND ANTIPSYCHOTIC INDUCED METABOLIC DYSFUNCTION

**DOI:** 10.1093/schbul/sby016.468

**Published:** 2018-04-01

**Authors:** Sarah Kanji, Ilona Gorbovskaya, Trehani Fonseka, Kazunari Yoshida, Victoria Marshe, Malgorzata MacKenzie, Premysl Bercik, Elena Verdu, Margaret Hahn, Daniel Mueller

**Affiliations:** 1 Centre for Addiction and Mental Health; 2 McMaster University; 3 University of Toronto

## Abstract

**Background:**

Antipsychotic (AP) medications are the cornerstone of treatment for schizophrenia (SCZ), with off-label prescription rapidly increasing in youth and adolescent populations. However, APs have been associated with metabolic side effects including diabetes and obesity. Although several mechanisms have been proposed, the gut microbiome (GMB) has been suggested as a potential mediator of AP-induced metabolic side effects due to its role in weight and metabolic regulation; as well as emerging evidence demonstrating a shift in the microbiome of AP-treated animals and humans. The purpose of the current study is to 1) Investigate the GMB in SCZ patients compared to healthy individuals and 2) To examine the role of GMB in SCZ and AP-induced metabolic side effects.

**Methods:**

Three groups of 25 participants are being recruited. Group A: Long-term AP-treated patients (for at least 6 months) taking clozapine (CLZ). Group B: Healthy controls matched with Group A for BMI, age, sex, and smoking status. Group C: Treatment-naive SCZ patients starting an AP or patients newly switching to CLZ. Groups A and B will be assessed at a single time point (week 0) whereas Group C will be assessed prospectively at weeks 0, 3, and 12 with the same measures collected. The following clinical measures and metabolic indices are collected at baseline, and if applicable, at follow up visits: the Questionnaire of Eating and Weight Patterns (QEWP), the Dutch Eating Behavior Questionnaire (DEBQ), the Power of Food Scale (POFS), the Positive and Negative Syndrome Scale (PANSS), the Gastrointestinal Symptom Rating Scale (GSRS), Exercise/Physical Activity Evaluation, body mass index, oral glucose tolerance test, lipid profile, and serum clozapine levels. We also collect fecal samples for DNA extraction and microbiome sequencing. We are currently performing a baseline comparison across groups A, B, and C in order to evaluate intrinsic differences between schizophrenia and healthy controls, as well as, effects of new AP exposure versus chronic exposure. The difference between analytical groups (i.e. groups A, B, and C) at different time points (i.e. baseline, week 3 and 6) for variables such as BMI and metabolic parameters marker change are analyzed using two-way repeated measures ANCOVA, including significant covariates. The relationship between different measures, such as body weight and metabolic parameters, GMB composition (abundance of each species), and AIWG will be tested using Spearman’s correlation. Analysis of the Operational Taxonomic Unit network generated using QIIME will be further analyzed using linear discriminant analysis (LDA) effect size (LEfSe) method. This method uses LDA scores to estimate the effect size of differentially abundant taxa (at phyla, class or other levels) and ranks the relative difference of microbial taxa that discriminate groups with biological consistency and statistical significance. The GMB composition of within group weight gainers versus non-weight gainer will also be assessed in a subgroup of subjects using LEfSe.

**Results:**

To date, 17 patients enrolled (13 patients in group A (9 men, mean±SD age: 31.6 ± 5.1 years; BMI, 31.4 ± 5.5), two control in group B (1 men, mean±SD age: 29.0 ± 1.4 years; BMI, 30.3 ± 0.8), two in group C (1 men, mean±SD age: 30.0 ± 9.9 years; BMI, 25.8 ± 5.6)). Further analyses including GMB composition in each group are now being conducted. We are currently analyzing these individuals for their GMB composition and results will be presented at the SIRS meeting.

**Discussion:**

To our knowledge, this is the first study to assess GMB sample in SCZ and investigate the association the GMB and AP-induced metabolic side effects. Our study has the potential to help to unravel the role of GMB in SCZ and AP response.

